# Experimental Analysis and Application of a Multivariable Regression Technique to Define the Optimal Drilling Conditions for Carbon Fiber Reinforced Polymer (CFRP) Composites

**DOI:** 10.3390/polym15183710

**Published:** 2023-09-08

**Authors:** Miguel Ángel Molina-Moya, Enrique García-Martínez, Valentín Miguel, Juana Coello, Alberto Martínez-Martínez

**Affiliations:** 1 Higher Technical School of Industrial Engineering of Albacete, University of Castilla-La Mancha, 02071 Albacete, Spain; miguelangel.molina4@alu.uclm.es (M.Á.M.-M.); enrique.gmartinez@uclm.es (E.G.-M.); 2 Regional Development Institute, Science and Engineering of Materials, University of Castilla-La Mancha, 02071 Albacete, Spain; alberto.martinez@uclm.es

**Keywords:** CFRP, delamination, drilling, image analysis

## Abstract

Carbon fiber reinforced polymers (CFRPs) are interesting materials due to their excellent properties, such as their high strength-to-weight ratio, low thermal expansion, and high fatigue resistance. However, to meet the requirements for their assembly, the drilling processes involved should be optimized. Defects such as delamination, dimensional errors and poor internal surface finish can lead to the premature failure of parts when bolt-joined or rivet-connected. In addition, the characteristic anisotropy and heterogeneity of these materials, and the issues related to the temperature reached during drilling, make it difficult to obtain optimal cutting parameters or to achieve high material removal rates. This research focuses on the optimization of the CFRPs drilling process by means of experimental analysis—varying the feed and spindle speed—for two different types of commercial drills—a twist tool and a dagger tool. An automatic image processing methodology was developed for the evaluation of the dimensional accuracy and delamination of the holes. The optimization was carried out using a multi-objective regression technique based on the dimensional deviations, delamination and surface finish. The areas with favorable machining conditions have been delimited for both tools and the results indicate that the twist tool allows one to achieve more productive cutting conditions than the dagger tool, when the combination of low feeds and high spindle speeds are the conditions to be avoided.

## 1. Introduction

Composite materials have gained widespread popularity in various industries due to their excellent mechanical properties and unique characteristics, such as their high strength, low weight, and corrosion resistance. Among these materials, carbon fiber reinforced polymers (CFRPs) stand out for their exceptional strength-to-weight ratio, making them ideal for high-performance applications where weight reduction is critical [1]. CFRPs are composed of a polymer matrix, typically an epoxy resin, reinforced with carbon fibers. The carbon fibers provide the material with excellent mechanical properties, including high tensile strength and stiffness, low thermal expansion, and excellent fatigue resistance [2]. As a result, CFRPs are commonly used in the aerospace, automotive, marine and other such industries.

In the aerospace industry, CFRPs are used to construct lightweight, high-performance components such as wings, fuselage sections, and engine nacelles [3]. The use of CFRPs in aerospace applications can result in significant weight savings, leading to improved fuel efficiency and increased range. In the automotive industry, CFRPs can be used in structural components such as chassis and body panels, leading to improved crashworthiness and reduced weight. Therefore, CFRPs are becoming increasingly important, replacing traditional materials and increasing production rates [4].

The machining of CFRPs is essential for the preparation of the manufactured parts prior to their assembly, with drilling being the most employed process [5]. However, the drilling of CFRPs can be challenging due to the material’s anisotropic and heterogeneous nature, which can result in issues such as delamination, poor surface quality, fiber fracture, fiber pull-out and rapid tool wear, among others [6]. These issues can lead to a reduction in the mechanical properties of the material, which can compromise the integrity of the final product. Moreover, the industrial productivity of the process is affected, leading to increments in manufacturing costs [7].

Delamination is a common defect in the drilling of CFRPs, causing separation of the layers, and can reduce the mechanical properties of the material. Delamination is caused by the separation of the bonded fiber layers, caused by an increase in stress above the bond limit due to cutting forces in the drilling process [8]. Delamination can occur either at the entry face of the hole, known as peel-up delamination, or at the exit side, known as push-down delamination [9]. It has been proven that the control of the delaminated surface around the hole is critical to ensure the mechanical strength of the joints made, such as the riveting of composite materials to metal parts. Increased delamination leads to stress concentration which adversely modifies the failure phenomenon [10]. Recently, Li et al. [11] have analyzed the effect of delamination on the compression properties of CFRPs by means of numerical simulation. They found that strength is remarkably affected by the delamination area but, if it is below a critical value or if delamination is placed in the middle of the hole, the compression properties are not affected. The characterization of this quality defect is not easy due to the wide range of geometrically dissimilar cases. It has become accepted to evaluate this according to the definition of a delamination factor that relates both the area of the hole itself and the damaged area around the hole. Different non-destructive inspection techniques, such as X-ray [12] or the use of ultrasound and infrared thermography procedures [13] have been employed to detect and evaluate the damage. The comparative application of X-ray tomography and ultrasonic C-scans has revealed similar delamination evaluation results [14]. Arhamnamazi et al. [15] found that the internal boundary delaminated area revealed by X-radiography was higher than those observed optically in the entry and exit surface, although the general shape was similar. However, specific equipment is required to carry out the analysis by applying these kinds of methodologies and they can be difficult to automatize for the assessment of a high number of drilled holes. For those reasons, some techniques, based on digital image analysis of both the entry and exit faces of the holes, have been applied for their evaluation [16,17,18]. Since internal delamination detection is not available, the delamination evaluation on the sheet faces is the most common procedure in industrial practice. Recently, some research has demonstrated that digital image processing techniques allow the delaminated area from visible two-dimensional defects to be determined with good accuracy [19]. For their part, Lukács et al. [20] have developed a novel image treatment algorithm that recognizes changes induced by the drilling process by comparing initial and post-drilled images and by applying image differencing. However, some manual intervention is required in order to tune the filter thresholds.

Regarding the process parameters, feed rate has been reported to be more influential than cutting speed on the thrust force, which is closely related to delamination [21]. Growing the feed rate leads to an increment of this force and, consequently, favors delamination. On the other hand, the increment of the cutting speed increases the cutting temperature, which may cause the matrix to burn and cause a loss of bonding [22], although it has been found to be effective in delamination reduction. Working at low cutting speed increases the risk of fiber pull-out [23]. The high modulus and strength of the carbon fibers make them susceptible to fracture if they are not cut properly. Rao et al. [24] have reported that the pin bearing tensile strength is reduced if delamination increases, making the feed rate the most significant factor for drilling damage. Similar results demonstrate that the influence of spindle speed on residual tensile strength is almost negligible in comparison with the feed rate contribution [25].

To reduce the delamination phenomenon, several strategies have been employed to improve the drilling process of CFRPs [2]. One of these strategies is the use of support plates that have been tested on the entry and exit side of the part, but their performance varies with the number of drilled holes and implies the introduction of a new different material that might cause additional tool wear, mostly when metallic plates are employed [26]. The selection of the proper tool material, coatings and tool geometry has also been evaluated, with solid carbide drills being the most used as they offer high performance and durability [27]. However, due to the material’s properties, the drills are subject to wear and chipping. The high strength and stiffness of the carbon fibers, combined with the anisotropy of the material, lead to high tool wear and breakage, reducing the tool life and increasing the cost of the process. Nevertheless, other studies point out that flank wear is predominant, and that no chipping appears [28]. In summary, the wear mechanisms depend on the relationship between feed rate and cutting speed employed. As a result, the increment of tool wear implies a higher risk of delamination. Different coatings such as diamond [29], TiAlN/TiN PVD [30] or nano-coatings (CrAlN/a-Si_3_N_4_) [31] have been investigated. Although some authors agree that coating allows lower surface roughness to be obtained than for uncoated carbide tools, their effect on tool wear, temperature and delamination have not been sufficiently established.

Twist drills and dagger drills, specifically designed for this purpose, have been compared, although some additional research has been developed that analyzes other types of drills [32]. It has been reported that the reduction of the drill point angle is favorable for the reduction of the thrust force, which can lead to delamination control. Some authors have reported that smaller point angles strongly reduce the probability of burr formations and enhance the hole quality [33]. Nevertheless, this implies higher cutting tool lengths and operation times, also increasing instability [34]. Although dagger configuration usually seems to be more efficient, the technological parameters that make the process the most productive still have not been established. Furthermore, experimental results from other authors contradict the good performance of these tools. Xu et al. [35] compared a wide range of drill bit geometries and found that the dagger tool performed worse than the twist and brad tools in terms of hole quality. For these reasons, further research and comparison of tools and cutting conditions remains necessary until the optimal conditions are unequivocally determined. Lubrication has also been studied as a solution to reduce tool wear and limit delamination, seeking the effective reduction of friction and temperature in the cutting zone. Cryogenic lubrication with liquid nitrogen, LN2, has been analyzed and some contradictory results have been found. Some authors agree that it leads to increases in the hardness and Young’s modulus of CFRPs and promotes brittleness, which results in higher thrust forces [36]. However, although some studies report that cryogenic lubrication leads to a worsening of the delamination zone [37,38], others defend the idea that low temperatures allow the reduction of delamination and internal hole surface roughness, achieving high qualities [36]. The impact of a minimum quantity of lubrication (MQL) on the drilling process of CFRPs has also been researched. It has been found that MQL is a promising technique for the reduction of tool wear, but it is not effective to reduce delamination [39].

Therefore, the development of machining analysis to develop efficient strategies by which to improve the drilling process and mitigate delamination defects is crucial for the successful manufacturing of CFRP components, allowing for it to become feasible to join parts. This article aims to analyze the effect of the technological parameters, that is, feed rate and cutting speed, and the type of solid carbide tool used, on the performance of the carbon fiber reinforced laminates drilling process in terms of delamination factor, interior hole surface roughness and dimensional precision. An experimental methodology based on digital image analysis of the delamination effect and a mathematical procedure, consisting of the multivariable regression and cubic surface spline fitting, is applied to obtain the most efficient factors combination.

## 2. Materials and Methods

### 2.1. Experimental Details

**Figure 1:**
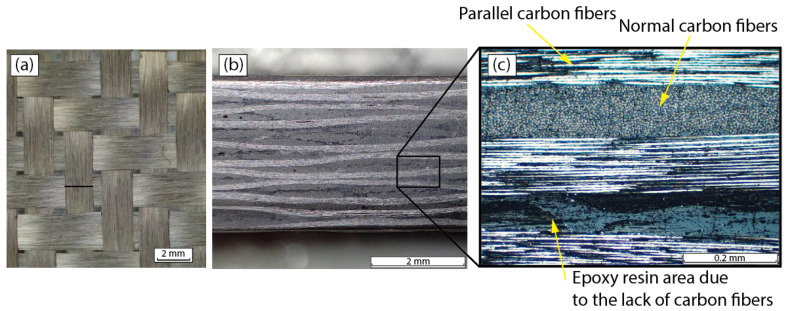
(**a**) Detail of 2 × 2 twill 3k woven carbon; (**b**) carbon fiber reinforced polymer, laminated and manufactured; and (**c**) detail of 0/90° oriented fibers.

The material used in this study was a carbon fiber reinforced epoxy (CFRP) laminate composite of 250 mm × 130 mm × 3.8 mm, Figure 1, manufactured with 12 plies of 2 × 2 twill 3k woven carbon, TC203T 3K (Toho Tenax Europe GmbH, Wuppertal, Germany), with a density of 200 g/m^2^ and a thickness of 0.25 mm, as shown in Figure 1a. The matrix materials used were commercial epoxy resin 1050 and hardener 1056 (Resoltech, Rousset, France) in a 100/35 ratio. The fiber volume fraction of the material was 48%.

The laminate was manufactured by manual contact and the polymerization and curing process was carried out at room temperature for 24 h. After, a post-curing treatment was applied at 80 °C for 4 h. Due to the definition of 2 × 2 twill of the fabric employed, some particularities may be pointed out about the introduction of the resin through the interlayer cavities. Thus, as can be seen in Figure 1b,c some areas of great coincidence of parallel fibers, 0°, and/or normal fibers, 90°, exist. Conversely, there are areas with the opposite configuration, i.e., where the long distance between fiber layers promotes the existence of thick films of resin. The local lack of fibers is justified properly by the constitutive waves of the twill and/or the incremental displacement of the fabric layers between them.

Details of the properties of each material employed can be found in Table 1.

**Figure 2:**
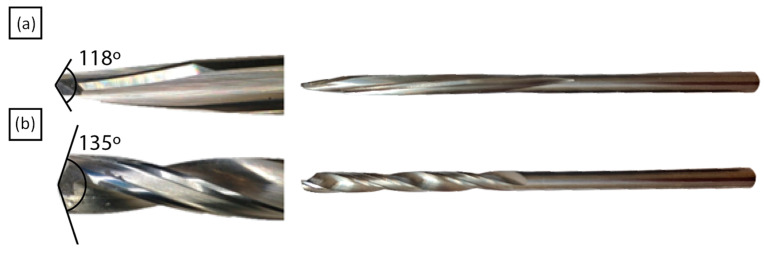
Tools used for drilling experiments: (**a**) dagger drill and (**b**) twist drill.

Two different types of uncoated 4.8 mm diameter drills were used during the tests, a twist drill 452.1-0483-044A0-CM H10F, and a dagger drill 452.1-0483-044A0-C H10F, from Sandvik Coromant (Sandviken, Sweden). These tools are suitable for the drilling of CFRPs according to the manufacturer. The drills had different geometries, as detailed in Figure 2.

**Figure 3:**
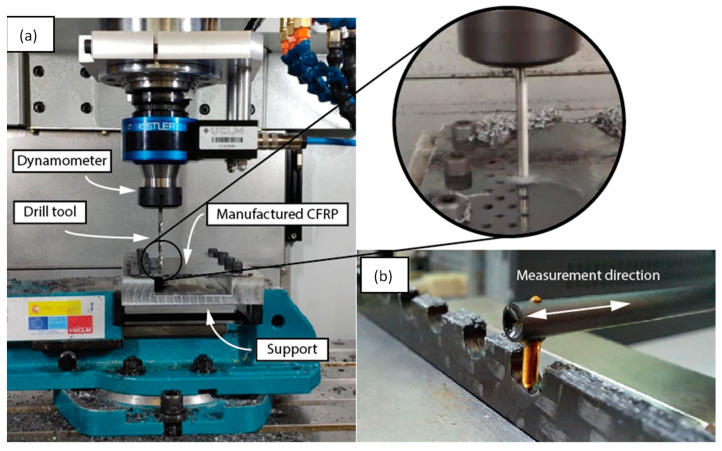
(**a**) Experimental drilling setup and (**b**) roughness measurement procedure.

The drilling tests were carried out on a LAGUN L850 CNC (Lagun Machine Tools, Ltd., Gipuzkoa, Spain) high speed machining center, as per Figure 3a. Dry drilling tests were carried out at three different feed rates of 0.01, 0.03 and 0.06 mm/rev and three spindle speeds of 1700, 6800 and 10,000 rpm, with each type of drill. The spindle speed range was chosen with the objective of achieving wide cutting speed intervals, i.e., of 25, 100 and 150 mm/min. Five holes were replicated for each machining condition. The thrust force involved was measured by using a Kistler 9170A rotary dynamometer (Kistler Instrumente AG, Winterthur, Swiss). The data acquisition frequency was adjusted to obtain 15,000 values during each test, meaning that it became a function of the feed rate. In order to position the composite in the machining center, an aluminum fixture was designed to permit an adequate work area while avoiding any deflection of the plate. Holes were staggered, with enough of a distance between them to avoid any machining interference. A tool wear test was undertaken for each tool, consisting of the drilling of 90 holes under the worst machining conditions experimented herein. As no significant wear was found, it was established that we were able to undertake the drilling experiments with only one tool, which may be considered as a new tool condition for each operation.

The drilled samples were cut through the middle of the holes diameter to measure the 3D and 2D roughness of the transversal machined surface in the drilling feeding direction. A Taylor Hobson Talysurf 50 contact profilometer (Taylor Hobson, Ltd., Leicester, UK) was used and the roughness analysis was carried out using the software Talymap Gold (Taylor Hobson Ltd., Leicester, UK), as shown in Figure 3b.

Five operations were undertaken for every machining condition to obtain all of the output variables with suitable repeatability.

### 2.2. Image Analysis Procedure for the Delamination Evaluation

**Figure 4:**
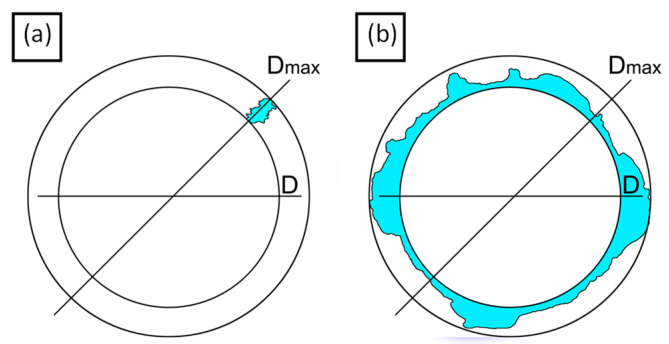
Delamination factor: (**a**) punctual delamination and (**b**) a general distributed delamination. Equation (1) results in the same *F_D_* value, while Equation (2) permits the delamination to be evaluated in a meaningful way.

To measure the delamination after the machining tests, pictures of the entry and exit of every hole were taken. One possibility avenue by which to evaluate the delamination is that according to the one-dimensional delamination factor, *F*_1*D*_, i.e., the ratio between the maximum diameter of the delaminated zone around the hole, *D_max_*, and the average diameter of the hole, *D*, according to Equation (1), Figure 4.

(1) $$F_{1D}=\frac{D_{max}}{D}$$

However, Equation (1) has some limitations; for example, there is no discrimination between holes that have the same maximum diameter, but different damaged areas. To solve this, a two-dimensional delamination factor should be used, as stated in Equation (2), where $F_{D}$ is defined as a two-dimensional factor, $A_{d}$ is the damaged zone area, and $A_{0}$ is the area of the theoretical hole.

(2) $$F_{D}=\left(\frac{A_{d}}{A_{0}}\right)\%$$

**Figure 5:**
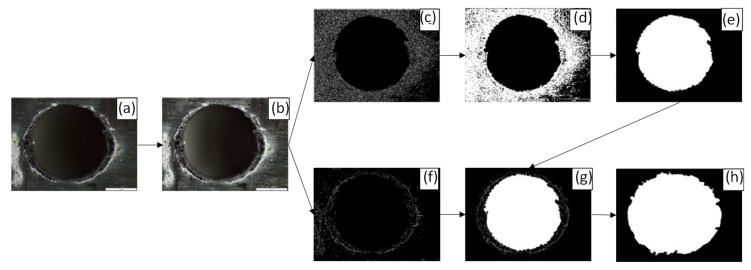
Image treatment methodology: (**a**) base image, (**b**) pretreated image, (**c**) canny output, (**d**) filled canny output, (**e**) hole area, (**f**) Prewitt output, (**g**) outliers removal and (**h**) delaminated area.

F_D_ evaluation requires a complete description of the whole delaminated perimeter. Thus, a code implemented in Matlab (The MathWorks, Inc., Natick, MA, USA)was developed to measure the delamination areas by image treatment, as shown in Figure 5. 

**Figure 6:**
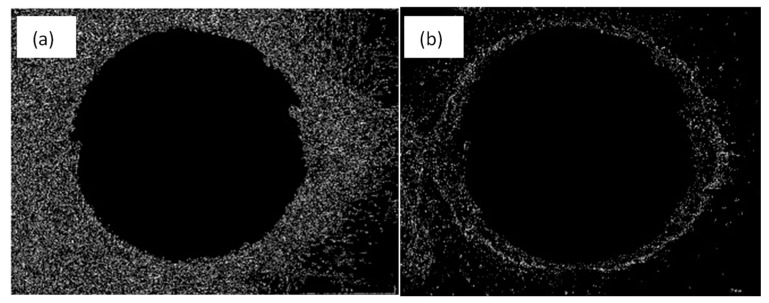
Results of the edge detecting algorithms. (**a**) Canny filter and (**b**) Prewitt filter.

The first step consists of preprocessing the image. In this phase, a Gaussian filter is applied to remove the possible noise that the microscope lens introduces. After that, a sharpen filter is employed to enhance the image to be analyzed with the edge detection algorithms, as shown in Figure 5b. The sharpen filter is a high-pass spatial filter that boosts high frequencies, increasing the definition of the picture using the command ‘imsharpen’ of Matlab. Border detection algorithms, such as edge (image, ‘Canny’), as in Figure 5c, and edge (image, ‘Prewitt’), in Figure 5f, were used to detect the image edges. The first algorithm permits a focus on the diameter and the second marks the delamination in a better way, Figure 6.

After filtering the images with the canny operator, the areas without interest are removed, particularly, the outer area of the hole. For that, a filling algorithm has been created by the authors in order to use the ‘bwboundaries’ function of Matlab^®^ to define the diameter border, as shown in Figure 5d,e. Finally, the value of the hole diameter can be automatically measured with the function ‘circfit’ from Andrew Horchler [40]. The radius value and the center coordinates of the hole are expressed in a number of pixels. Thus, it was necessary to establish the correlation between the length-units and the number of pixels. 

Considering the center coordinates and radius derived from the ‘circfit’ operator, a new enlarged circle is created. This circle is added to the Prewitt output, as in Figure 5f, using ‘imadd’, obtaining a total definition of the resulting image. Additionally, the hole area defined by the Canny filter output is also added, as in Figure 5g. The differences can be seen in Figure 5f,g, in which the pixel outliers are thrown out. 

The commands ‘imdilate’ and ‘imerode’, along with ‘imfill’, are applied to the Prewitt output, resulting in the delamination area, as in Figure 5h. In this way, a correct definition of the image is constructed in order to apply the ‘bwboundaries’ operator to quantify delamination.

### 2.3. Statistical Response Surface Generation and Standardization of the Variables Involved

**Figure 7:**
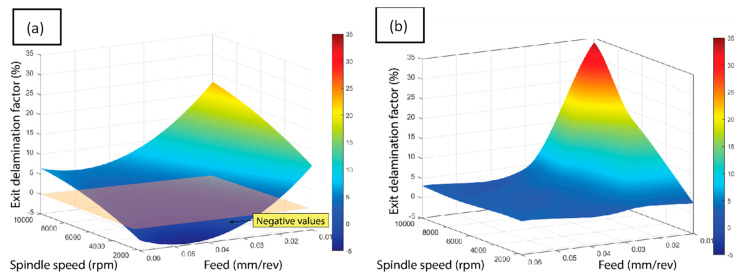
(**a**) ANOVA response surface showing an anomalous trend and (**b**) MATLAB’s cubic spline surface.

For the analysis of the experiments, the response surface methodology (RSM) was implemented, by establishing the relationship between the input parameters (spindle speed, feed rate, drill geometry) and the measured outputs (entry and exit delamination, entry and exit diameter deviation, and Ra). An early ANOVA analysis showed that the correlation of the involved variables led to low suitability of this technique. Thus, the RSM was implemented using MATLAB’s ‘Curve Fitting Toolbox’, which fits a cubic spline surface between the experimental data. This procedure applies an approximated polyline that takes into consideration the influence of the different correlation points, as shown in Figure 7. With this method, a total of five response surfaces were obtained.

The polysurfaces permitted the estimation of the values of the output variables, discretizing the range of the input variables into 200 × 200 points. For the corresponding analysis, this system can be assimilated into a 200 × 200 ordered matrix [M_i_], where *i* = 1 to 5 according to the five output variables. Each position in the matrices is noted as *m_j,k_,* where the subindex *j* corresponds to the feed and the *k* subindex represents the spindle speed. As the absolute values of the different output variables have different magnitudes of order it is recommended to normalize them in order to balance the influence of the output variables in a multi-objective optimization. The normalization process consists of comparing each estimated output value with the objective value, equaling zero in all cases, and dividing the result by the maximum absolute value examined for the variable, $\mathrm{Max}\left|m_{j,k}^{i}\right|$. Thus, every element *n_j,k_* of the normalized matrix of each variable *i*, *[N_i_]* is obtained according to Equation (3) and their values are between 0 and 1.

(3) $$n_{j,k}^{i}=\frac{m_{j,k}^{i}-\;0}{\mathrm{Max}\left|m_{j,k}^{i}\right|}$$

All of these normalized matrices are then added with a ponderation factor, *f_i_*, according to the relevance established for each output variable in the multi-objective optimization. The result is an optimization matrix, *[O]*, Equation (4), from which the optimal drilling parameters can be obtained by selecting the element of *[O]* with the minimum value. In this case, all output variables are considered with the same ponderation weight, that is, *f_i_* equals 1 for all cases.

(4) $$[O]=\sum_{i=1}^{i=5}f_{i}\left[N_{i}\right]$$

**Figure 8:**
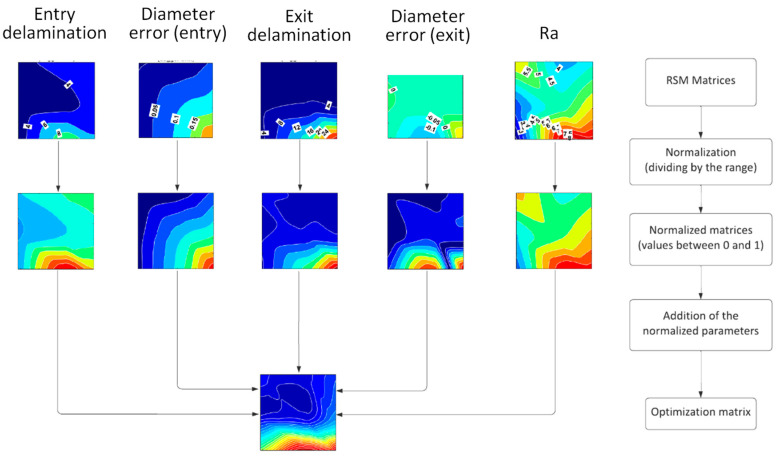
Methodology for obtaining the optimized matrix composed by the RSM individual matrices corresponding to the output variables.

The methodology considered to obtain the optimization matrix, *[O]*, is depicted in Figure 8.

## 3. Results and Discussions

### 3.1. Cutting Forces

**Figure 9:**
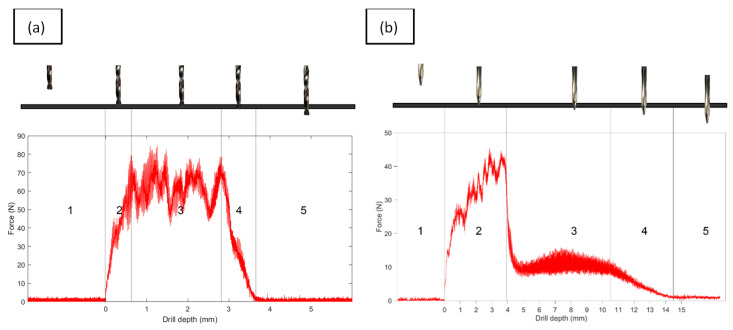
Thrust force registers of the drilling operations: (**a**) twist drill and (**b**) dagger drill.

In Figure 9, the typical registers of thrust drill forces with both of the used tools are shown. We can observe the different evolution of the forces for both tools employed. This difference is due to the conical tip of the dragger tool that, as expected, makes the initial penetration of the tool smoother and promotes a generally consistent force during the drilling operation. However, some more machining time is necessary in order to examine the total depth of the drilled holes. 

**Figure 10:**
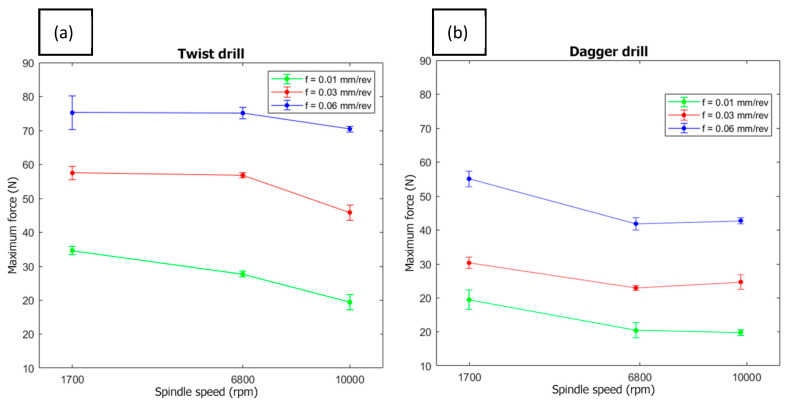
Thrust force as a function of the feed rate and the spindle speed: (**a**) twist drill and (**b**) dagger drill.

The measured forces in the tests are depicted in Figure 10. It is observed that, among the input parameters, the feed rate and the tool geometry heavily influence the thrust force that appears in the machining process. The dagger drill reduces the thrust force up to 35% when compared with the twist drill. The feed rate produces an increment in the thrust force, due to the higher material removal rate. On the other hand, an increment in the cutting speed only produces a slight decrement in the force. This can be explained if it is taken into consideration that the cutting speed increases the temperature on the cutting zone, which can in turn soften the epoxy resin, i.e., the matrix of the composite material. This behavior has been found by other authors [19], although these authors found a lower influence of the cutting speed than that obtained herein, as they worked with a cutting speed range narrower than that under investigation here. 

Nevertheless, the low value of the thrust force is not significant in the process and will not be considered an output variable for the drilling operation optimization.

### 3.2. Delamination and Dimensional Accuracy

**Figure 11:**
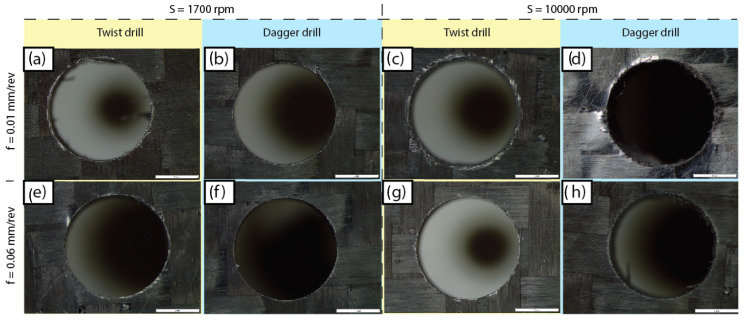
Entry surface delamination for different experimental conditions.

**Figure 12:**
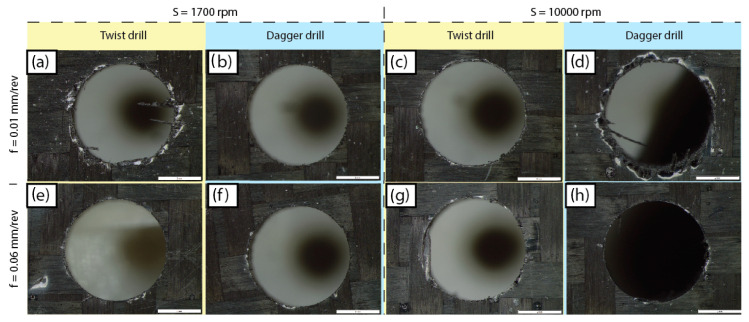
Exit surface delamination for different experimental conditions.

In Figure 11 and Figure 12 different drilled surfaces are collected and the hole quality on the entry and exit surfaces, respectively, can be observed. 

Clearly, the quality of the hole at the entry face is better than the quality at the exit face. This can be explained according to the lower stiffness of the composite in the exit zone. In the entry, the drill starts cutting a plate of 3.8 mm thickness. However, when the last plies are cut, the lack of support under the plate leads to the push-down delamination effect being worsened.

Comparing the different cutting conditions, delamination is more frequent at 0.01 mm/rev of feed rate for both drills. This is due to the increased time required to complete the hole and the low heat transfer capacity of the composite material, which could increase the amount of heat retained in the cutting zone, in turn leading to matrix softening and its deformation before the cut. Temperature increment in the hole area due to the reduction of the feed rate has been previously reported by Marques et al. [4].

**Figure 13:**
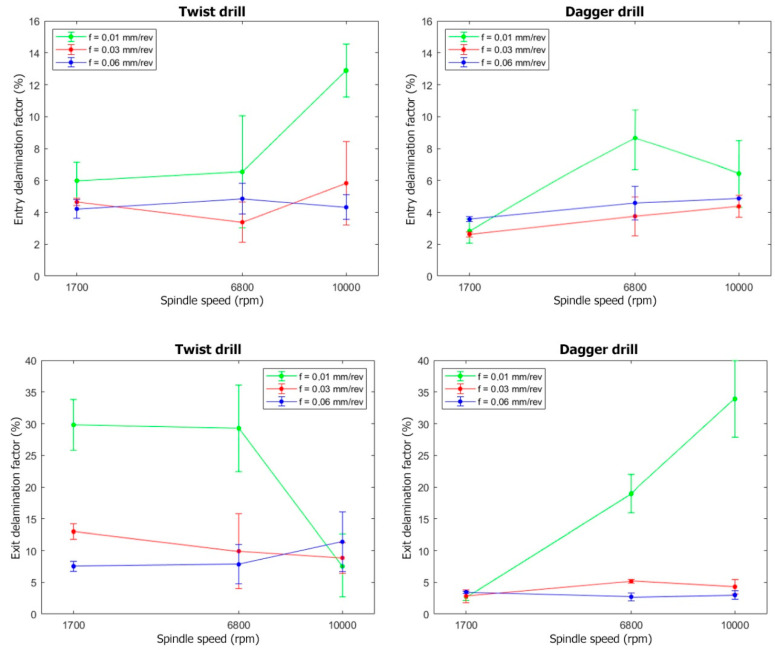
Entry and exit delamination factor for the twist and dagger drills at different cutting conditions.

From the developed image analysis methodology, two indicators of the hole quality are extracted—the average diameter and the delamination factor—both in the entry and the exit of each hole. The results are presented in Figure 13.

The delamination factor is worse when drilling at 0.01 mm/rev, compared with the other feed rates. This phenomenon is noticeable when the dagger drill is used, as the delamination factor is extremely high compared with the others. With such low feed rates, a minimal cross-section is cut, so the drill bit is not able to stabilize itself and starts to vibrate. The chatter phenomena effect is observed in the exit of the hole, shown in Figure 12d, leading to the higher delamination factor in Figure 13. On the other hand, the delamination factor obtained for the dagger drill is always lower than those quantified for the twist drill if the 0.03 and 0.06 mm/rev feed rates are applied.

Furthermore, if 0.03 or 0.06 mm/rev feed rates are used, the cutting speed does not show a significant effect, finding similar results from 1700 to 10,000 rpm for both tools. According to these results, the lower feed rate of 0.01 mm/rev should be avoided, as it does not permit an increase to the cutting speed, and thus leads to the lowest machining removal rates.

**Figure 14:**
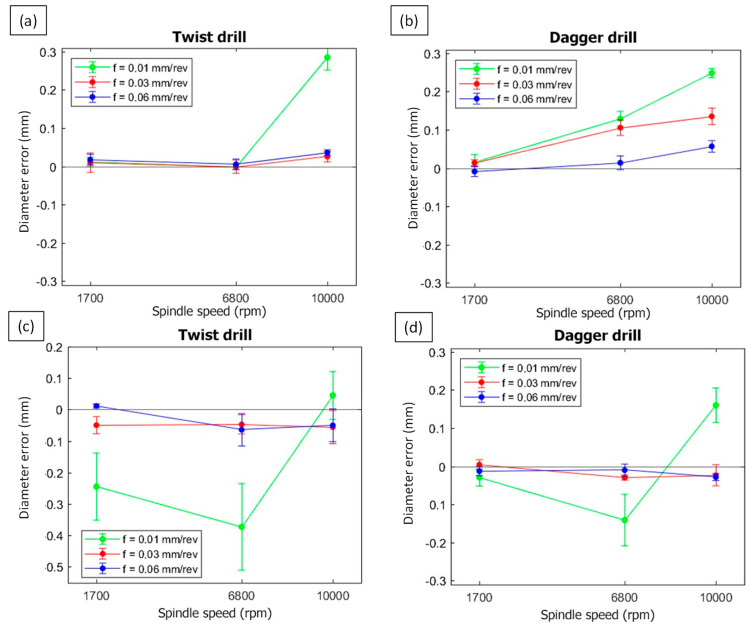
Entry (**a**,**b**) and exit (**c**,**d**) diameter errors of the drilled holes for the twist and dagger drills and different cutting conditions.

Figure 14 shows the diameter error for the entry and exit faces. Similar to the delamination results, the worst cases are found for the lowest feed rate (0.01 mm/rev) and highest cutting speed if the entry surface is considered. The errors at the entry are always positive, which means that the hole diameter is greater than the nominal diameter of the drill. This oversizing trend, wherein the cutting speed is increased, generates higher cutting temperatures and leads to thermal expansion in the matrix, has been widely reported in the literature [38]. In particular, a feed rate of 0.01 mm/rev presents the worst conditions with respect to the diameter error. 

However, negative values are reported for the exit diameter errors, and these are caused by the push-down delamination and burrs. The delaminated fibers cover part of the hole, leading to detection errors in the image treatment analysis methodology. For the algorithm, both the hole size and push-down fibers behave as dark indistinguishable areas, resulting in a limitation of the automated procedure. This means that an analysis of the diameter error cannot be carried out because some anomalous results should be obtained. Nevertheless, although the diameter error will not be evaluable in the exit zone, this parameter provides additional information on the state of the delamination of the hole.

### 3.3. Surface Finishing Analysis

**Figure 15:**
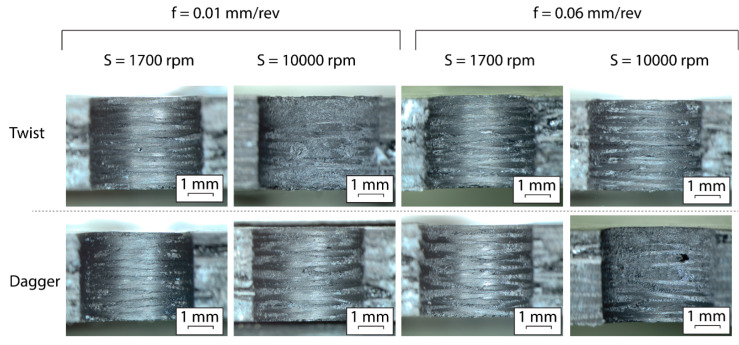
Macroscopic photographs corresponding to different drilling conditions with the twist and the dagger tools.

**Figure 16:**
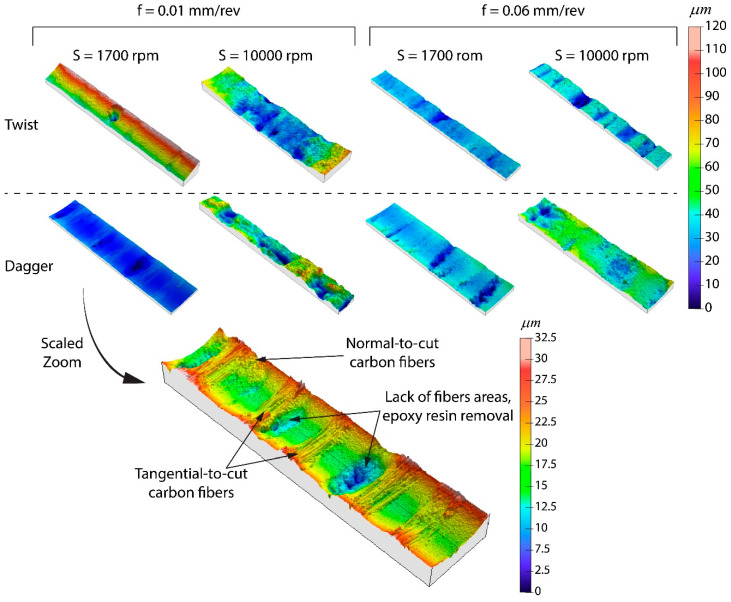
Three-dimensional surface maps corresponding to central bands, 250 μm wide, of the inner surfaces of selected holes.

In Figure 15 micrographs corresponding to the hole surface at different machining conditions are shown. It has sought to select, as the most representative, those belonging to the largest speed and feed rate values. One can observe the layers of the composite, while some pores and empty long thin areas are also able to be observed. Moreover, some pull-out areas can appear (i.e., dagger, 10,000 rpm, 0.06 mm/rev). Finally, some plateaus corresponding to each fiber layer can be distinguished. All of these features are confirmed in the 3D surface maps shown in Figure 16 that were measured on a central band, 250 μm wide, of the surfaces shown in Figure 15.

According to Figure 16 the best finishing conditions correspond to a drilling spindle speed of 1700 rpm as the involved surfaces present a smooth topography. Nevertheless, the case involving the rate of 0.01 mm/rev and the twist tool belongs to this class as the topography is strongly conditioned by a punctual pull-out phenomenon. The worst finishing surfaces were obtained for 10,000 rpm and 0.01 mm/rev, which confirms the theory wherein the softening of the resin justifies the delamination behavior. Thus, the areas of the resin without fiber tend to be pulled out and easily worn away, leading to the valleys observed in the 3D profiles. 

**Figure 17:**
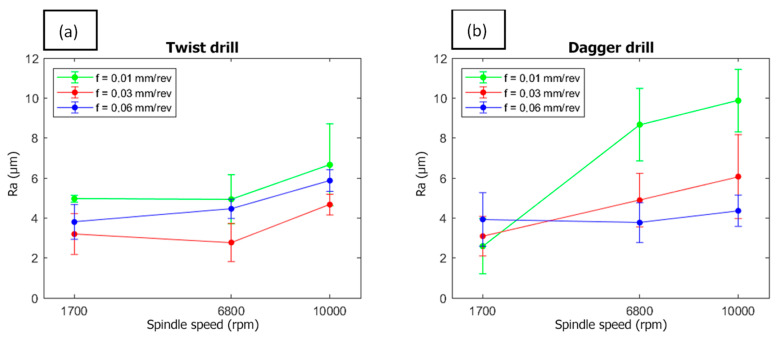
Ra values of internal drilled holes surfaces; (**a**) twist drill and (**b**) dagger drill.

A quantitative analysis was carried out from 2D arithmetic roughness, Ra, by measuring five samples corresponding to each drilling condition. In Figure 17 the Ra results are shown and agree with those presented before for the involved outcomes. Nevertheless, regarding the 3D profiles, some punctual issues may significantly modify the 2D roughness parameters. Thus, for both tools, higher Ra values are obtained for larger spindle speeds. For the twist tool, the best finishing corresponds to 0.03 mm/rev, while 0.06 mm/rev is the best condition for the dagger tool. In any case, 0.01 mm/rev has to be avoided, particularly for the dagger tool, an observation which is coherent with the delamination and the diameter accuracy found.

### 3.4. Optimization

**Figure 18:**
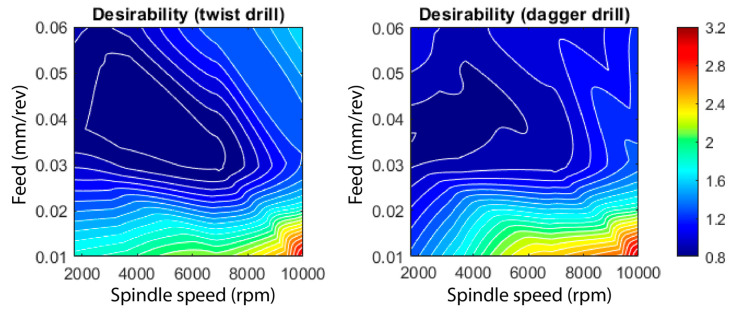
Multi-objective response surface corresponding to the twist and dagger drills.

Figure 18 depicts the response surfaces obtained according to the methodology mentioned above. Initially, all of the parameters measured during the tests were considered. However, the exit diameter error was eventually removed from the general analysis as the results offered were not conclusive. Thus, the response surfaces represent the sum of the entry and the exit delamination factors, the entry diameter error and Ra. Therefore, the range of the possible value is 0–4, with 0 being the best expected result and 4 the worst.

As can be deduced from Figure 18, the tendency observed is similar for both tools, with the worst conditions corresponding to high spindle speed and low feed rate. The stability area of the machining conditions is marked by the dark blue color, which involves a range of spindle speed up to 8000 rpm and feed rates between 0.03 and 0.06 mm/rev. Nevertheless, the twist tool permits higher productivity machining conditions to be reached. Moreover, it can be stated that the experimental conditions selected in this work are suitable since a feed rate larger than 0.06 mm/rev tends to give worse results. In this context, only for a narrow band of the spindle speed, would a higher feed rate permit acceptable results to be obtained. Finally, of the best machinability areas shown in Figure 18, the output results become worse and faster, and the unexpected influence of the low feed rate values must be pointed out.

**Figure 19:**
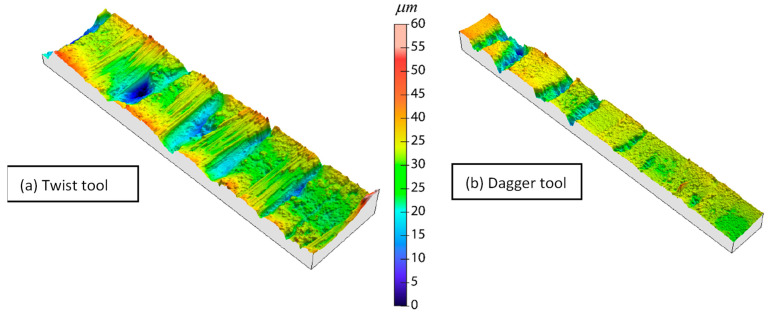
Three-dimensional finishing surface under optimal drilling conditions: (**a**) twist tool and (**b**) dagger tool.

The optimum conditions correspond to 0.05 mm/rev and 4000 rpm, and 0.04 mm/rev and 1700 rpm, for the twist and dagger tools, respectively. Table 2 indicates the comparison between the experimental results obtained after machining under the optimal conditions and those predicted by the corresponding response surfaces under those conditions. It can be observed that there is an excellent agreement regarding the dimensions and delamination of the entry diameter. The exit delamination presents some differences that can be justified based on the fabric pull-out phenomenon, as it has a more chaotic behavior, as has been established. Something similar happens if Ra is considered, although the results remain acceptable. Again, under optimal drilling conditions, the random localization of the fiber-empty-resin areas may significantly alter the Ra value, as shown in Figure 19.

## 4. Conclusions

An image analysis methodology has been developed that permits us to measure the delamination and dimensions of the holes automatically and without the need to tune them beforehand. The methodology only uses the functions and filters of Matlab^®^.

Through the analysis of the data from the tests, and the optimization process that has been carried out, the following conclusions can be extracted:

- The fabric configuration of the CFRP analyzed herein significantly influences its behavior. Fiber-lacking areas promote the resin peel-out that affects the hole surface quality.
- The delamination phenomenon is larger at the exit face of the hole due to the low rigidity of the existing material at the end of the operation. The shape of the exit delamination is more chaotic and makes it difficult to analyze the exit dimensions of the hole diameter.
- It has been determined that low feed rate values should be avoided as they generate a heating effect on the resin matrix that promotes its softening and favors the involved defects. A combination of medium-to-high feed rates and low-to-medium spindle speeds make up the working area.
- The optimal value that leads to the best combination of the output parameters (entry and exit delamination, entry diameter accuracy, and roughness) for each tool that we investigated has been established. The values obtained, 4000 rpm and 0.05 mm/rev for the twist tool and 1700 rpm and 0.04 for the dagger tool, demonstrate that the twist tool exhibits a more productive behavior.

## Figures and Tables

**Table 1:** Carbon fiber and epoxy resin properties.

Carbon Fiber		Epoxy Resin	
Density (g/cm^3^)	1.77	Density (g/cm^3^)	1.10
Tensile strength (MPa)	4100	Flexural strength (MPa)	116
Tensile modulus (GPa)	240	Flexural modulus (GPa)	3.35
Elongation (%)	1.7	Elongation (%)	7.7

**Table 2:** Experimental vs. predicted output variables under optimal drilling conditions.

Output Variable	Twist Tool			Dagger Tool		
	Experimental	Predicted	Error (%)	Experimental	Predicted	Error (%)
Entry diameter (mm)	4.85	4.83	0.41	4.82	4.84	0.41
Entry *F_D_* (%)	3.45	3.38	2.07	2.19	2.51	12.75
Exit *F_D_* (%)	8.67	7.87	10.17	3.13	2.31	35.49
Ra (µm)	4.53	3.4	33.26	3.5	3.7	5.40

## Data Availability

The data used to support the findings of this study may be shared upon request.
